# Enhanced neutrophil phagocytic capacity in rheumatoid arthritis related to the autoantibodies rheumatoid factor and anti-cyclic citrullinated peptides

**DOI:** 10.1186/s12891-015-0616-0

**Published:** 2015-06-30

**Authors:** Marcelo Bogliolo Piancastelli de Siqueira, Licia Maria Henrique da Mota, Shirley Claudino Pereira Couto, Maria Imaculada Muniz-Junqueira

**Affiliations:** Laboratory of Cellular Immunology, Pathology, Faculty of Medicine, Campus Darcy Ribeiro, Asa Norte, University of Brasília, Brasília, DF 70.910.900 Brazil; Rheumatology Service, University Hospital of Brasília, Faculty of Medicine, University of Brasília, Brasília, DF 70.910.900 Brazil

**Keywords:** Rheumatoid arthritis, Anti-cyclic citrullinated peptide antibodies, Rheumatoid factor, Phagocytosis, Oxygen radicals

## Abstract

**Background:**

There is no consensus on the mechanisms by which anti-cyclic citrullinated peptide antibodies (anti-CCP) and rheumatoid factor (RF) influence the pathogenesis of rheumatoid arthritis (RA). The current study verified if the presence of RF or anti-CCP is associated with phagocytic capacity and reactive oxygen species (ROS) production by phagocytes in RA patients to better clarify the role played by these antibodies in pathogenesis of the disease.

**Methods:**

A cohort of 30 RA patients followed from early stages of the disease were characterized by positivity for RF or anti-CCP, disease activity score (DAS-28), health assessment questionnaire (HAQ), use of synthetic or biologic therapy, lifestyle, comorbidities and radiographic erosions. Phagocytic capacity against *Saccharomyces cerevisiae* and superoxide anion production were assessed in RA patients and compared with 20 healthy controls. Phagocytic capacity and superoxide anion production were also compared between RF- and anti-CCP-positive and -negative RA patients.

**Results:**

Anti-CCP- and RF-positive RA patients had higher neutrophil phagocytic capacity than anti-CCP- (*p* = 0.005) and RF (*p* = 0.005)-negative individuals through pattern-recognition receptors. As assessed via pattern recognition or opsonin receptors, neutrophils and monocytes from RA patients presented overall higher phagocytic capacity than neutrophils and monocytes from healthy controls (*p* < 0.05). Furthermore, RA patients also showed a higher capacity for producing cytotoxic oxygen radicals (*p* = 0.0026). Phagocytosis and superoxide anion production did not correlate with any of the clinical variables analyzed in this study.

**Conclusions:**

This study showed increased phagocytosis by neutrophils in RA patients who were positive for anti-CCP and RF autoantibodies. Furthermore, there was an overall hyperactivation of the phagocytes in RA patients. Our data suggest that anti-CCP and RF may indirectly enhance the inflammation cascade involving neutrophils and may indirectly sustain tissue damage in RA. Targeting the production of these autoantibodies may be a promising strategy in the management of RA.

## Background

Recent breakthroughs in understanding of the pathogenesis of rheumatoid arthritis (RA) have led to advances in the diagnosis and management of the disease [1–3]. The inflammatory process seems to begin with the recognition of arthritogenic antigens by CD4^+^ T helper (Th) lymphocytes followed by macrophage and fibroblast stimulation [4]. Cytokine production by these cells and Th17 cells drive the inflammatory process. Regulatory T cells may not exhibit adequate regulatory activity in RA, possibly due to impaired production of interleukin (IL)-10 and transforming growth factor (TGF)-β [1, 3].

The initial trigger for RA seems to be the post-translational citrullination of extracellular synovial proteins that often occurs during innocent episodes of inflammation in predisposed individuals. The newly citrullinated proteins are able to generate a B cell response that results in the production of anti-citrullinated protein antibodies. These antibodies may enter the joints and promote local inflammation and tissue destruction [5]. It has been suggested that phagocytes play a central role in synovitis by locally releasing cytokines and reactive oxygen species (ROS) [6].

Anti-citrullinated cyclic peptide antibodies (anti-CCP) and rheumatoid factor (RF) are associated with more severe disease characterized by earlier onset, faster progression, more extra-articular manifestations and worse outcome [7]. Bacterial cell wall components can induce RF synthesis by B cells during infections in patients with a predisposed genetic background [8]. However, there is no consensus on the mechanisms by which anti-CCP and RF influence the pathogenesis of the disease, and the role played by these antibodies in RA pathogenesis remains unclear.

The relationship between the presence of anti-CCP and RF and the severity of disease in RA is well established [1, 7, 9, 10]. The presence of these autoantibodies can potentially trigger an imbalance in the innate immune response leading to enhanced phagocytic capacity and production of ROS. This imbalance caused by anti-CCP and RF may account for the greater severity of the disease. However, the role of the functional state of phagocytes in RA is controversial. Phagocytosis has been shown to be increased [11, 12], decreased [13] or not significantly different [14, 15] in neutrophils of RA patients compared to those of healthy individuals. In addition, higher levels of anion superoxide production were observed in RF positive patients compared to RF negative patients [11]. However, it is still unclear whether the presence of anti-CCP or RF influences the phagocytic capacity and ROS production of neutrophils and monocytes in patients with RA. The aim of this study was to determine the state of activation of phagocytes in RA patients and the relationship with RF and anti-CCP autoantibodies. We compared phagocytic capacity and ROS production between patients with RA and healthy individuals. We further compared phagocytic capacity and ROS production between subsets of patients who were positive or negative for anti-CCP or RF. This knowledge may broaden understanding of the pathophysiological mechanisms involved in RA that are dependent on innate immunity and autoantibodies.

## Methods

### Ethical issues

The ethical rules of the actualized Helsinki Declaration and the Brazilian National Council of Health for experimentation in human beings were strictly followed. The Human Research Ethical Committee of the School of Medicine of the University of Brasília approved the experimental protocol (process number 048/2010), and each volunteer gave written informed consent for blood donation.

### Subjects

One author (LMHM), a rheumatologist, conducted clinical evaluations of rheumatic and control individuals and screened 30 individuals from the Brasília Cohort of Rheumatoid Arthritis for enrollment in this study. These patients had been followed in the rheumatology outpatient clinic at Brasília University Hospital since diagnosis of early RA [16–19]. Early RA was defined as the occurrence of joint symptoms consistent with the disease for a minimum of six weeks and a maximum of 12 months. Every patient retrospectively fulfilled the EULAR/ACR 2010 criteria [20] and was evaluated for innate immune function after 36 months follow-up in this study.

Patients received the recommended standard treatment regimen, including traditional disease modifying anti-rheumatic drugs (DMARDs) or biological therapy as needed. RA treatment followed the Brazilian Society of Rheumatology instructions [21, 22].

Demographic and clinical data, including age, time since diagnosis, disease activity index (Disease Activity Score 28 – DAS28) [23], functional incapacity (Health Assessment Questionnaire – HAQ) [24], use of synthetic or biologic DMARDs (medication, dosage), lifestyle (physical activity, current or prior smoking), education and comorbidities were assessed through questionnaires and review of medical records.

The control group, which comprised 20 healthy adult volunteers without rheumatic disease and without a personal or familial history of rheumatic disease, was evaluated to provide comparative baseline values for phagocytosis and ROS production in normal healthy individuals.

The exclusion criteria for both groups included any condition capable of influencing the immune system, such as cancer, diabetes, infectious disease, recent surgeries or any drug with direct influence on the immune system except for those used to treat RA.

RF and anti-CCP2 autoantibodies were detected from blood samples collected at the beginning of the follow-up period. Samples were stored and sent to INOVA Diagnostics, Inc. (San Diego CA, USA) where RF and anti-CCP2 were tested using Quanta Lite™ RF IgM ELISA and Quanta Lite™ CCP IgG ELISA (INOVA Diagnostics, CA, USA), respectively, according to the manufacturer’s protocol. For RF, values greater than 15 IU/mL were considered positive. For anti-CCP2, values greater than 20 U were considered positive.

Radiographs of the hands, wrists, feet, and ankles were performed. Erosions were defined as localized loss of definition of cortical bone near the capsular insertions or as areas of bone lysis encompassing cortical and cancellous bone. To avoid the effects of incidental findings of isolated erosions on the analysis, only patients with a minimum of two erosions in two distinct articulations were classified as erosive. Two independent observers (a radiologist and a rheumatologist) evaluated the radiographs at the end of the study, and erosions were considered present only when there was agreement between the two observers.

### Phagocytosis test

Testing for phagocytosis of *Saccharomyces cerevisiae* was adapted from Muniz-Junqueira et al. [25]. Briefly, 40 μl samples of heparinized blood from each subject were placed on glass slides containing eight marked areas of 7-mm diameter each. Samples were prepared in duplicate and incubated in a wet chamber for 45 min at 37 °C. The slides were rinsed with 0.15 M phosphate-buffered saline (PBS), pH 7.2 at 37 °C to remove non-adherent cells and incubated for 30 min with a suspension of 6.25 × 10^4^ non-sensitized or sensitized *S. cerevisiae* in 20 μl Hanks-Tris solution (Sigma, St. Louis, MO, USA), pH 7.2. Non-sensitized *S. cerevisiae* was incubated with 10 % inactivated fetal calf serum (Invitrogen, Carlsbad, CA, USA), whereas, sensitized *S. cerevisiae* was previous incubated with 10 % fresh serum from the control or RA donor individual. Slides were rinsed with PBS to remove non-phagocytosed *S. cerevisiae*, fixed with absolute methanol and stained with 10 % Giemsa solution. The number of *S. cerevisiae* ingested by 200 neutrophils and 200 monocytes in individual preparations was assessed by optic microscopy. The phagocytic index (PI) was calculated as the mean yeast cell intake per phagocytizing neutrophil or monocyte multiplied by the percentage of these cells engaged in phagocytosis [26]. *Saccharomyces cerevisiae* (Baker’s yeasts) suspensions were prepared according to a previously described technique [25] to assess phagocytosis via pattern-recognition receptors and facilitated by opsonins. When *S. cerevisiae* are prepared by this technique and incubated with human complement from human fresh serum, it retains considerable C3 activity on its surface [25, 27–29]. By previous standardization, it was observed that the ingestion of the particles sensitized by fresh serum occurs preferentially through complement receptors, with about 300 % decrease in the phagocytic index of monocytes by using sensitized yeasts before and after inactivation of complement at 56 °C [25]. In addition, the presence of human immunoglobulins adsorbed to yeast cells was detected by immunofluorescence [25].

The internalization of particles by phagocytes occurs via receptors. When phagocytosis occurs via pattern recognition receptors, the phagocyte recognizes directly conserved pattern molecular on the surface of the particle to be phagocytosed. When phagocytosis is facilitated by opsonins, the ingestion occurs via receptors to components of complement or to FcγIgG (CR1, CR3 and FcR) in neutrophils/monocytes that will recognize their respective ligands during the process of phagocytosis [25, 30, 31].

In this work, yeasts were used with or without previous incubation with fresh serum from the donor. In the former case, yeast cells were considered sensitized, because they were opsonized by complement molecules, particularly C3, and immunoglobulin molecules present in fresh serum. These molecules adhere on *S. cerevisiae* surface and will be recognized by their neutrophil or monocyte receptors (CR1, CR3 and FcγIgGR) during the process of phagocytosis [25, 29, 32, 33]. Whereas, yeast cells that were incubated with heat inactivated fetal calf serum were considered as non-sensitized, because they were non-opsonized and their phagocytosis occurs via the pattern-recognition receptors (PRRs) of neutrophils or monocytes [25, 29, 32, 33]. For opsonization, the *S. cerevisiae* prepared as referred above were sensitized by incubation at 37 °C for 30 min with 10 % fresh serum from the donor in Hanks-Tris solution. The non-opsonized yeast cells were incubated with 10 % heat inactivated fetal calf serum for 30 min at 37 °C. They were non-sensitized, and their phagocytosis occurred via the pattern-recognition receptors of phagocytes [25, 29, 32, 33].

### Nitro blue tetrazolium slide test

The nitro blue tetrazolium (NBT) test was adapted from Muniz-Junqueira et al. [34]. This test evaluates the ability of phagocytes to generate toxic oxygen radicals capable of reducing the compound NBT to an insoluble form, called formazan. Formazan can be identified under optical microscopy by a blue color in the cytoplasm of the cell. The amount of NBT reduced is directly proportional to the amount of ROS produced by phagocytes [34]. Briefly, phagocytes adhered to slides were incubated with 0.05 % NBT in a suspension of 6.25 × 10^4^ sensitized *S. cerevisiae* in 20 μl Hanks-Tris solution for 20 min at 37 °C in a humidified chamber. The slides were washed, fixed with methanol and stained with 1.4 % safranin and 28.6 % glycerol in distilled water. The percentage of phagocytes with reduced NBT in the cytoplasm was quantified using optical microscopy.

### Statistical analysis

The Kolmogorov-Smirnov test for normality of distribution and Bartlett’s test for equal variance were applied to variables before comparative analysis. For non-normal distributions, the Mann Whitney non-parametric statistic test was employed to compare two unrelated samples. For normal distributions, the *t* test was employed. For samples with normal distributions but different variances, the *t* test was adjusted using Welch’s correction. Data are expressed as medians, quartiles and extreme values. Differences with a two-tailed p value of < 0.05 were considered statistically significant. The Prism® 5.0 software package (GraphPad Software, Inc., San Diego, CA, USA, 2005) was used for analysis and graphical design of the data.

## Results

### Clinical, laboratory, radiographic and therapeutic characteristics

Data from the 30 RA subjects enrolled in the study are summarized in Table 1. In the RA group, subjects were predominantly female (28 patients, 93 %), with a mean age ± SD of 50.47 ± 9.9 years. The control group was composed of 20 healthy individuals, 19 of whom were women (95 %) with 1 man (5 %) (*p* = 1.0, Fisher’s exact test). The mean age ± SD for the control groups was 38.9 ± 2.1 years (*p* = 0.001, Mann–Whitney test).

**Table 1:** Clinical and laboratory characteristics of patients with RA evaluated in Brasília University Hospital (n = 30)

Characteristics	RA (total)	Anti-CCP-	Anti-CCP+	RF-	RF+	*p*- value
	(*n* = 30)	(*n* = 9)	(*n* = 21)	(*n* = 12)	(*n* = 18)	
Gender (%)						
Women	28 (93.3 %)	8 (89 %)	20 (95 %)	11 (92 %)	17 (94 %)	>0.05*
Men	2 (6.66 %)	1 (11 %)	1 (5 %)	1 (8 %)	1 (6 %)	
Age (years)	50.47 ± 9.9	51.6 ± 10.2	50 ± 10.1	52.3 ± 10.4	49.3 ± 9.7	>0.05^#^
Time since diagnosis (years)	6 ± 2.8	5 ± 2.9	6 ± 2.4	6 ± 2.3	6 ± 1.8	>0.05^+^
Education (years)	7.9 ± 2.9	6.6 ± 1.4	8.5 ± 3.1	8.0 ± 3.2	7.9 ± 2.5	<0.05^##^
Physical activity	12 (40 %)	5 (55.6 %)	7 (33.3 %)	6 (50 %)	6 (33.3 %)	>0.05**
Smoking (%)	7 (23.3 %)	2 (22.2 %)	5 (23.8 %)	2 (16.7 %)	5 (27.8 %)	>0.05*
Treatment (%)						
Synthetic DMARD	21 (70 %)	7 (77.7 %)	14 (66.6 %)	9 (75 %)	13 (72.2 %)	>0.05*
Biologic DMARD	9 (30 %)	2 (22.20 %)	7 (33.3 %)	3 (25 %)	5 (27.7 %)	>0.05*
DAS 28	3.47 ± 0.5	3.7 ± 0.3	3.36 ± 0.6	3.6 ± 0.5	3.4 ± 0.5	>0.05^+^
HAQ	0.84 ± 0.4	0.9 ± 0.4	0.8 ± 0.4	0.9 ± 0.4	0.8 ± 0.4	>0.05^+^
Radiographic erosions at initial evaluation (%)	16 (53.3 %)	2 (22.2 %)	14 (66.6 %)	5 (41.7 %)	13 (72.2 %)	<0.05**

*Chi-square; ^#^ *t* test; ^##^ *t* test with Welch’s correction (anti-CCP- < anti-CCP+); + Mann–Whitney test; **Fisher’s exact test (anti-CCP+ > anti-CCP-)

When these patients were included in the Brasília Cohort, the mean period of duration of articular symptoms was 27 ± 15.6 weeks, with 10 patients (33 %) having less than 12 weeks of symptoms. Eleven patients (36.7 %) had not received any treatment for RA prior to the initial evaluation. The mean follow-up, from the time of diagnosis until blood was collected to evaluate phagocytosis and ROS production, was 6 ± 2.8 years (Table 1).

Twenty-one out of 30 (70 %) RA patients were positive for anti-CCP, and 18 out of 30 (60 %) were positive for RF. Among the patients positive for anti-CCP, 17 (81 %) were strong positives, and 4 (19 %) were weak positives (Table 1).

Ten patients (33.3 %) were on non-steroidal anti-inflammatory medications, and six patients (20 %) were using prednisone (<10 mg/day). Seventy percent of patients (21/30) were using synthetic DMARDs and 30 % (9/30) were on biological therapy. Seventy percent of patients on synthetic therapy were on a combination therapy (methotrexate plus leflunomide, methotrexate plus hydroxychloroquine or methotrexate plus sulfasalazine), and 30 % were on monotherapy (methotrexate or leflunomide). All patients on a biologic therapy were also on methotrexate, except for one patient. Patients on methotrexate received 5 mg/week folic acid supplementation.

A high percentage of patients (25/30 patients – 83.3 %) had at least one comorbidity associated with RA. The main morbid conditions found were arterial hypertension (22/30 – 73.3 %), fibromyalgia (16/30 – 53.3 %), dyslipidemia (10/30 – 33.3 %), depression (8/30 – 26.6 %), anxiety disorders (7/30 – 23.3 %), hypothyroidism (11/30 – 36.6 %) and osteoporosis (2/30 – 6.6 %).

### Phagocytosis through pattern recognition receptors (PRR)

#### Neutrophils from individuals with RA showed higher phagocytosis rates than healthy controls, which was a consequence of an increase in phagocytosis that occurred in anti-CCP and RF positive individuals

**Fig. 1:**
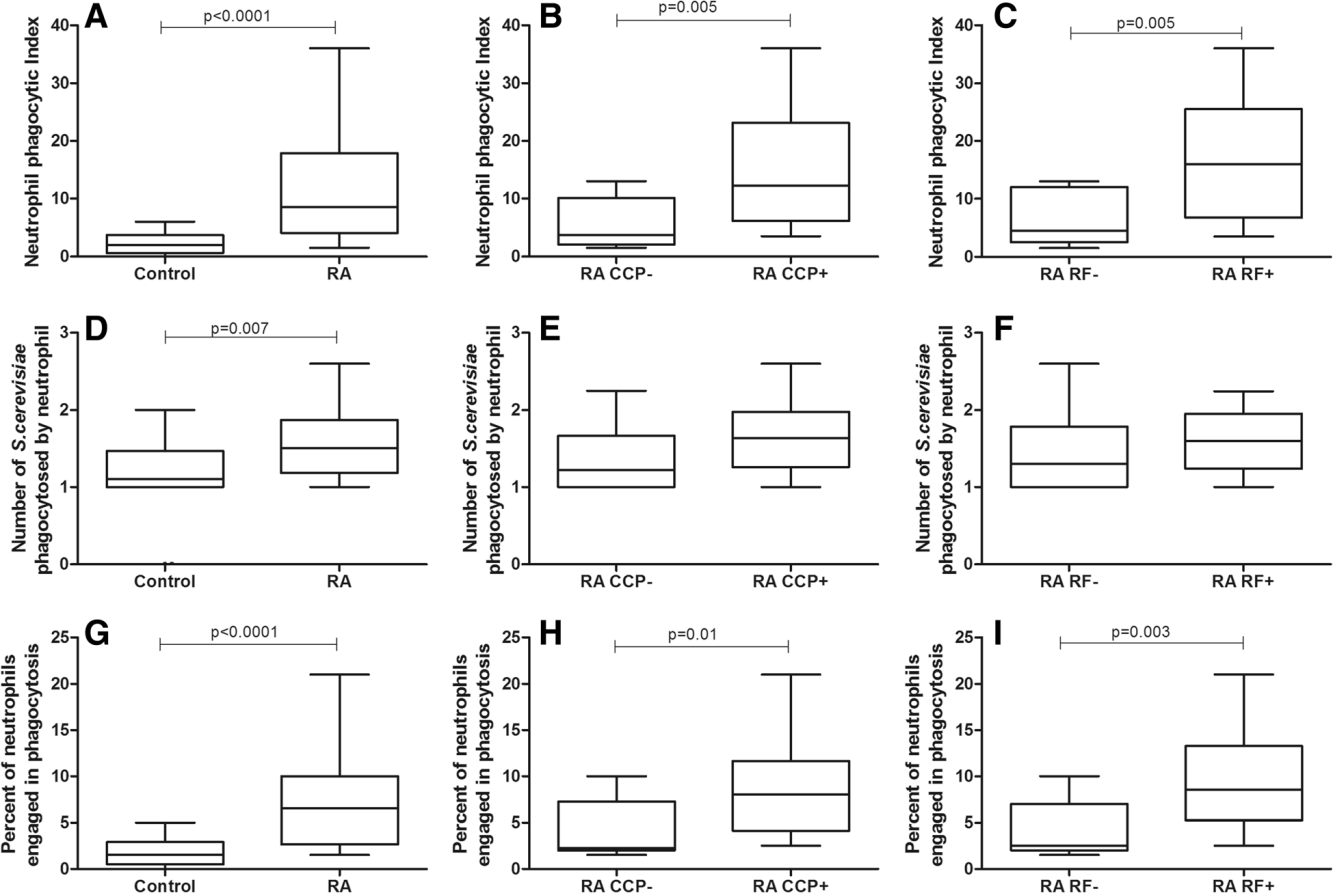
Effects of anti-CCP and RF positivity on phagocytosis of non-opsonized yeast cells by neutrophils from RA patients. Data are expressed as medians, quartiles and extreme values. RA caused a rise in the phagocytosis of non-opsonized yeast (*p* < 0.0001; *t* test with Welch’s correction) and anti-CCP-positive and RF-positive individuals displayed a higher phagocytic index. This increase was due to an increase in the percent of neutrophils engaged in phagocytosis, but no difference was observed in the number of yeast ingested between anti-CCP- or RF-negative or -positive individuals (*p* > 0.05). Top: neutrophil phagocytic index (**a**, **b**, **c**); Middle: average number of S. cerevisiae yeast cells ingested by phagocytosing neutrophil  (**d**, **e**, **f**); Bottom: percent of neutrophils engaged in phagocytosis (**g**, **h**, **i**). The statistical differences are marked in the graphics and in text

To verify whether there was a difference in neutrophil phagocytosis between RA and control individuals, the phagocytic index (PI) of individuals with RA was compared with that of healthy control individuals. Using non-sensitized *S. cerevisiae*, neutrophils from individuals with RA had a PI that was 4.25 times higher than that of healthy controls (8.5 vs. 2.0, respectively, *p* < 0.0001, *t* test with Welch’s correction, Fig. 1a). This increase was caused by a higher engagement of phagocytes in phagocytosis (*p* < 0.0001, *t* test with Welch’s correction, Fig. 1g) and by a higher median number of phagocytosed *S. cerevisiae* per neutrophil (*p* = 0.007, Mann–Whitney test, Fig. 1d) in RA patients compared to healthy control individuals.

Comparison of anti-CCP negative and positive RA patients showed a higher PI in the group that was positive for the antibody (*p* = 0.005, Mann–Whitney test, Fig. 1b). Similar results occurred when comparing RF negative and positive RA patients. Those patients with a positive RF showed a higher phagocytic index than RF-negative patients (*p* = 0.005, Mann–Whitney test) (Fig. 1c). The increase in PI was caused by higher engagement of neutrophils in phagocytosis in both cases (*p* = 0.01, Mann–Whitney test, for anti-CCP positive, Fig. 1h; and *p* = 0.003, Mann–Whitney test, for RF positive, Fig. 1i).

#### Higher phagocytosis by monocytes from RA patients in contrast with healthy controls. Positivity to autoantibodies was not associated with higher phagocytosis

**Fig. 2:**
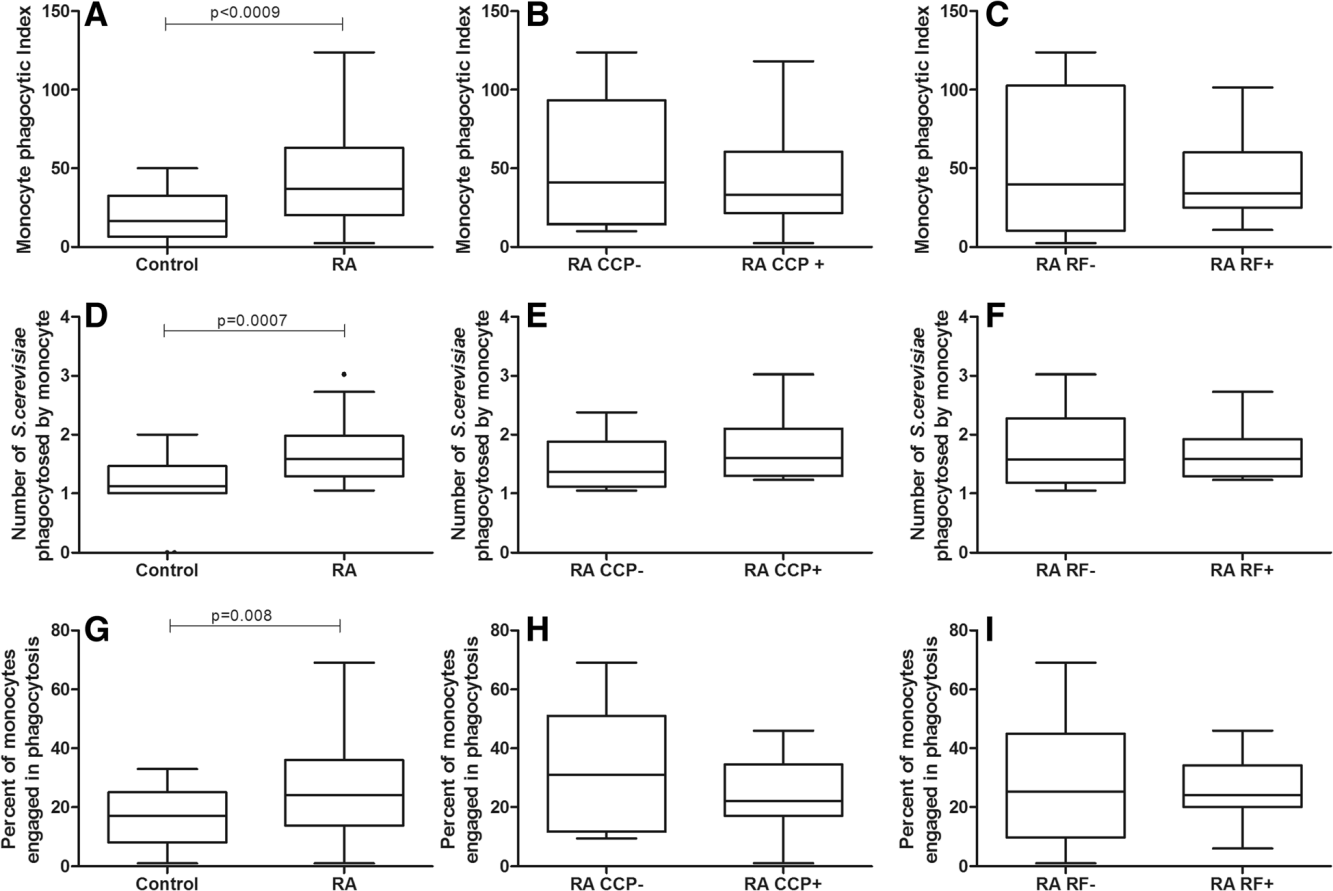
Effect of anti-CCP and RF positivity on the phagocytosis of non-opsonized yeast cells by monocytes from RA patients. Data are expressed as medians, quartiles and extreme values. RA caused a rise in the phagocytosis of non-opsonized yeasts (*p* < 0.0009; *t* test with Welch’s correction), but no difference was observed between anti-CCP- or RF-negative or -positive individuals (*p* > 0.05). Top: monocyte phagocytic index (**a**, **b**, **c**); Middle: average number of S. cerevisiae yeast cells ingested by phagocytosing monocyte (**d**, **e**, **f**); Bottom: percent of monocytes engaged in phagocytosis (**g**, **h**, **i**). The statistical differences are marked in the graphics and in text

The monocyte PI was 2.28 times higher in RA patients (36.75) compared to the control group (16.15) (*p* = 0.0009, *t* test with Welch’s correction, Fig. 2a). This result occurred due to both increased yeast cell intake (*p* = 0.0007, Mann–Whitney, Fig. 2d) and higher monocyte engagement in phagocytosis (*p* = 0.008, *t* test, Fig. 2g). However, there was no significant difference when comparing the monocyte PI between anti-CCP-negative or -positive individuals (*p* > 0.05, Figs. 2b, e, h) and between RF-negative or -positive individuals (*p* > 0.05, Fig. 2c, f, i).

### Phagocytosis through opsonins

#### Neutrophils from individuals with RA showed higher phagocytosis through opsonin receptors than healthy controls. Autoantibody positivity was not associated with higher phagocytosis

**Fig. 3:**
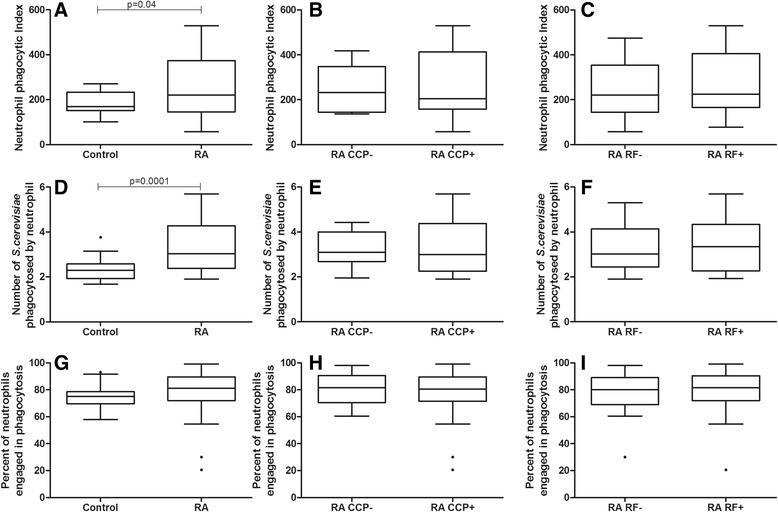
Effect of anti-CCP and RF positivity on the phagocytosis of opsonized yeast cells by neutrophils from RA patients. Data are expressed as medians, quartiles and extreme values. RA caused a rise in the phagocytosis of opsonized yeast (*p* < 0.04; Mann–Whitney test), but no difference was observed between anti-CCP- or RF-negative or -positive individuals (*p* > 0.05). Top: neutrophil phagocytic index (**a**, **b**, **c**); Middle: number of S. cerevisiae yeast cells phagocytosed by neutrophil   (**d**, **e**, **f**); Bottom: percent of neutrophils engaged in phagocytosis (**g**, **h**, **i**). The statistical differences are marked in the graphics and in text

The neutrophil PI in RA patients (220) was 1.3 times higher than the neutrophil PI in the control group (169) (*p* = 0.04, Mann–Whitney test, Fig. 3a). This difference occurred due to increased yeast cell intake only (3.0 vs. 2.3, *p* = 0.0001, *t* test with Welch’s correction, Fig. 3d), as there was no significant difference in the proportion of neutrophils engaged in phagocytosis (*p* = 0.16, Mann–Whitney test, Fig. 3g). No difference was observed when comparing the PI in subgroups of RA patients who were negative or positive for anti-CCP (*p* = 0.82, Mann Whitney test, Fig. 3b) or RF (*p* = 0.62, *t* test, Fig. 3c).

#### Monocytes from RA patients showed higher phagocytosis rates than healthy controls. No difference was observed between anti-CCP- or RF-negative and -positive individuals

**Fig. 4:**
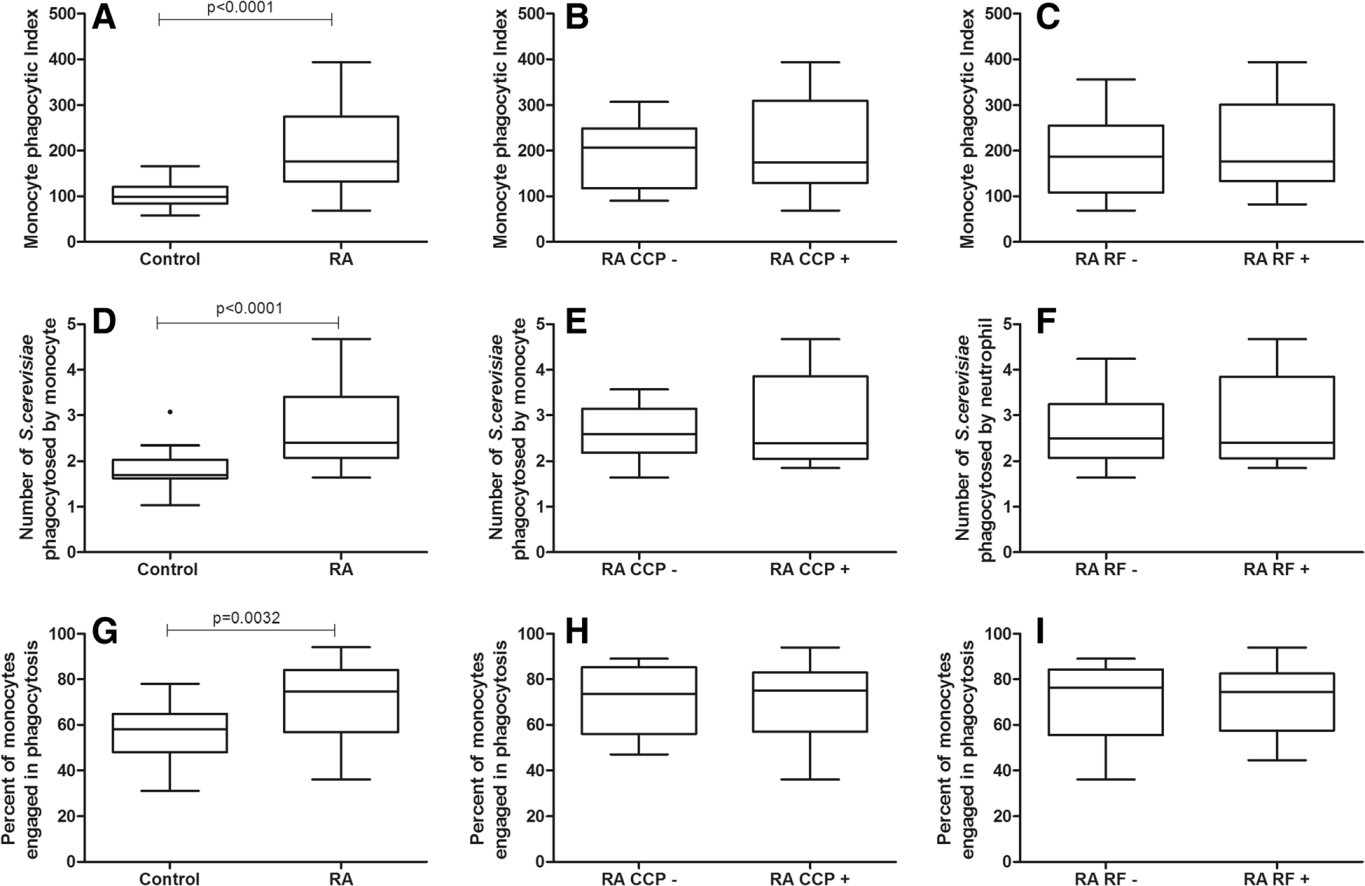
Effect of anti-CCP and RF positivity on the phagocytosis of opsonized yeast cells by monocytes from RA patients. Data are expressed as medians, quartiles and extreme values. RA caused an increase in the phagocytosis of opsonized yeast (*p* < 0.001; *t* test with Welch’s correction), but no difference was observed between anti-CCP- or RF-negative or -positive individuals (*p* > 0.05). Top: monocyte phagocytic index (**a**, **b**, **c**); Middle: number of S. cerevisiae yeast cells phagocytosed by monocyte (**d**, **e**, **f**); Bottom: percent of monocytes engaged in phagocytosis (**g**, **h**, **i**). The statistical differences are marked in the graphics and in text

The median PI of monocytes assessed through the use of opsonin receptors was 1.79 times higher in RA patients (176.5) compared to the control group (98.8) (*p* < 0.0001, *t* test with Welch’s correction, Fig. 4a). This difference was due to both an increased proportion of monocytes engaged in phagocytosis (74.5 % vs. 58 %, *p* = 0.003, Mann Whitney test, Fig. 4g) and increased yeast cell intake (2.39 vs. 1.69, *p* < 0.0001, Mann Whitney test, Fig. 4d). There was no significant difference in PI when comparing anti-CCP-negative and -positive subgroups (*p* > 0.05, Fig. 4b, e, h) or RF-negative and -positive subgroups (*p* > 0.05, Fig. 4c, f, i).

### RA patients showed higher production of superoxide anion than healthy individuals

**Fig. 5:**
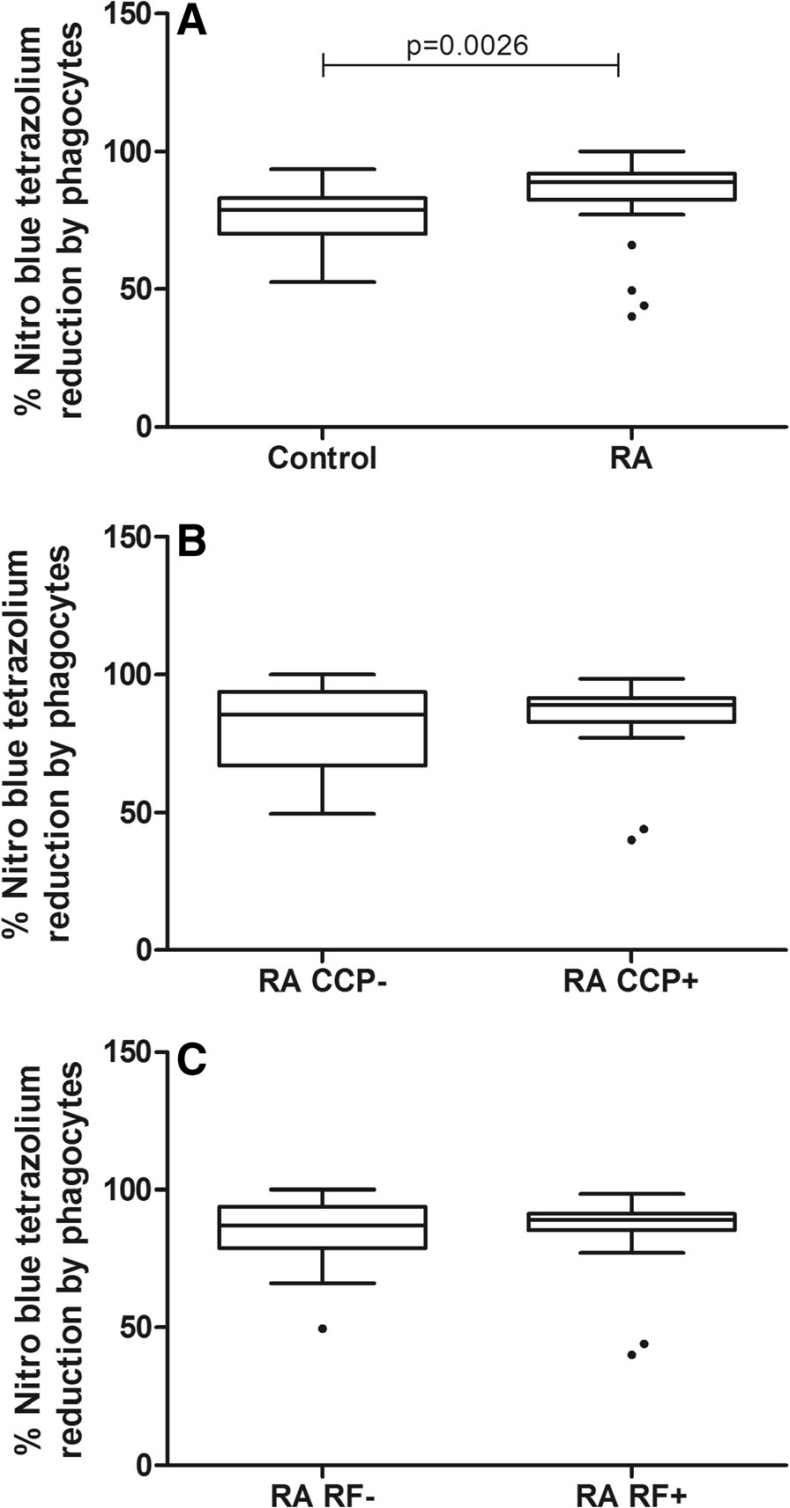
Effect of anti-CCP and RF positivity on the production of superoxide anions by phagocytes from RA patients. Data are expressed as medians, quartiles and extreme values, and outlier values are indicated. Individuals with RA produced a significantly higher amount of superoxide anion than controls (*p* = 0.0026, Mann–Whitney test). Positivity for anti-CCP or RF was not statistically associated with increased superoxide anion production (*p* > 0.05). **a**: rheumatoid arthritis patients versus healthy control; **b**: anti-CCP negative versus positive RA patients; **c**: RF negative versus positive RA patients. The statistical differences are marked in the graphics and in text

Production of superoxide anions was evaluated using the NBT test. The percent reduction in NBT was significantly higher in RA patients (88.75 %) than in the control group (78.75 %) (*p* < 0.0026, Mann–Whitney test, Fig. 5a), which demonstrates a greater capacity to generate oxygen radicals. However, neither anti-CCP positivity (*p* = 0.82, Mann–Whitney test, Fig. 5b) nor RF positivity (*p* = 0.88, Mann–Whitney test, Fig. 5c) altered the percent reduction of NBT.

### No association between phagocyte functions of peripheral neutrophils and monocytes and the clinical, radiological and therapeutic characteristics analyzed was observed in this study

Although radiographic erosions at initial evaluation were more frequent in anti-CCP-positive individuals (Table 1), no association between phagocytosis or superoxide anion production by peripheral neutrophils and monocytes and the clinical, radiological, and therapeutic variables analyzed in this study were observed. These variables included disease activity (DAS28), functional incapacity (HAQ), use of synthetic or biologic DMARDs (medication, dosage), lifestyle (physical activity, current or prior smoking), education, comorbidities and presence of radiographic erosions (*p* > 0.05 for all, Mann–Whitney test).

## Discussion

This study showed an overall increased neutrophil and monocyte phagocytosis and superoxide anion production in RA patients. However, a relationship between enhanced phagocytosis and positivity for anti-CCP and RF was only observed when neutrophil phagocytosis was evaluated via pattern recognition receptors (Fig. 1). Neutrophils and monocytes from RA patients presented higher overall phagocytic capacity than healthy controls when assessed via both pattern recognition receptors and opsonin receptors. In addition, RA patients showed a higher capacity to produce cytotoxic oxygen radicals. Lastly, no association between phagocyte functions and superoxide anion production by neutrophils and monocytes and the clinical, radiological and therapeutic variables analyzed in this study was observed.

In contrast to our results, some authors showed normal [14, 15] or decreased [13, 35] phagocytic capacity of neutrophils or monocytes/macrophages in individuals with RA. However, similar to our results, Okuda et al. [11] and Paino et al. [12] also observed increased phagocytosis by neutrophils in RA patients. Some possibilities to explain these differences include differences in research protocol used, early *versus* advanced RA disease in study patients [14, 15, 35], and different criteria used to define the disease [13–15].

In our study, enhanced phagocytic capacity was repeatedly observed in both neutrophils and monocytes from RA patients. Similarly, this enhance capacity was observed in the analysis of phagocytosis via both PRR and opsonin receptors. The concordance of all of these results renders our data more reliable. In addition, the clinical criteria used to define RA were strictly followed in this study.

There are multiple possible mechanisms that may be suggested to explain the increase in phagocytosis via PRRs observed in RA patients. Our data showed that the increase in phagocytic capacity of monocytes and neutrophils was caused by an increase in the number of phagocytosed particles per phagocyte and by a higher engagement of phagocytes in phagocytosis. Enhanced expression of PRRs involved in uptake of the yeast in neutrophils and monocytes may explain the increased number of particles phagocytosed in RA disease. In fact, it has been shown that the scavenger receptor CD36 is up-regulated in THP-1 macrophages that are exposed to plasma from RA patients [36]. CD36 is a PRR that is expressed in phagocytes and mediates yeast uptake [25, 30, 31]. The over-expression of PRRs in phagocytes is most likely caused by overproduction of inflammatory cytokines, such as tumor necrosis factor (TNF)-α, which is enhanced in RA patients [1–3]. In addition, TNF-α is also known to enhance phagocytosis [37]. The fact that positivity for anti-CCP and RF only increased neutrophil phagocytosis via PRRs suggests that this increase was caused through an indirect mechanism, possibly through stimulation of cytokine production that enhanced PRR expression at the membrane surface of phagocytes.

To be able to phagocytose, macrophages need to move towards the particle. Because the increase in phagocytosis observed in this study was also caused by an increased number of neutrophils/monocytes involved in the process, one cannot refute the possibility that RA also caused an enhancement in the mobility of macrophages toward the particle.

Monocytes and neutrophils from RA patients also showed increased phagocytic capacity via opsonins. When phagocytosis occurs via opsonins, the particle is covered with complement components or immunoglobulin, and ingestion occurs via receptors to components of complement (mainly C3b, which is a ligand of complement receptor 3 (CR3)) [25, 30, 31] and/or via receptors to the Fc portion of IgG [30]. Immunofluorescence staining showed that both immunoglobulin and complement are present on the surface of sensitized yeast [25]. In fact, increased expression of CR3 in neutrophils recovered from the synovial fluid of patients with RA has been shown [38].

We suggest that the lack of difference between phagocytosis via opsonin receptors observed for anti-CCP-positive and -negative RA patients may have occurred because the antibody against CCP is of the IgG class and thus binds to an IgG receptor. In our system, the IgG receptor may be occupied by anti-CCP antibodies, leaving a lower number of receptors available for occupation by sensitized *S. cerevisiae*. In addition, the binding of anti-CCP to its IgG receptor should stimulate the phagocyte, enhancing phagocytosis and superoxide anion production, both of which we observed in this study.

RFs are IgM antibodies directed to the Fc fragment of IgG molecules [39]. It is possible that RF (IgM against IgG) bound to the Fc portion of the IgG molecule that was bound to the yeast prevented its interaction with the IgG receptor (Fcγr) on the phagocyte in our system. This situation would cause a decrease in the number of *S. cerevisiae* that were available to be phagocytosed.

In addition, indirect actions of anti-CCP and RF should also be considered. It has been shown that immune complexes containing citrullinated fibrinogen co-stimulate macrophages via Toll-like receptor 4 and the Fcγ receptor to enhance TNF-α production [40, 41], which strongly stimulates phagocytes. TNF-α is increased in RA patients, which reinforces this possibility [1–3]. Furthermore, activation of complement via both classical and alternative pathways by immune complexes containing anti-CCP [42] and RF may indirectly activate phagocytes.

It is unclear the different influence of antibodies anti-CCP and RF in phagocytosis by neutrophils and monocytes we observed. We suggest that this difference may have occurred because the subsets of monocytes that was evaluated in peripheral blood in this work. It has been shown that the non-classical Ly6C^−^ monocytes are required for the development of inflammatory arthritis in mice [43]. The non-classical monocyte phenotypes in human may show different responses and this monocyte accounts for only 10 % of all human blood monocytes [44].

Parallel to the observed increase in phagocytosis, RA patients also showed significantly increased superoxide anion production. Our data were similar to others that showed increased oxygen radical production in RA [45–47]. We were unable to detect differences in superoxide anion production between anti-CCP- or RF- positive and -negative RA patients. It is possible that because RA causes high O^−^ production, the differences between the two RA subgroups were difficult to detect. Conversely, Okuda et al. [11] showed higher superoxide anion production in individuals who were RF positive. It is possible that this discrepancy was due to differences in the protocol used, the severity of disease, and the duration of the disease for patients in the two studies.

Although it was not our primary objective, we attempted to evaluate whether other variables, such as disease activity (DAS28), functional incapacity (HAQ), use of synthetic or biologic DMARDs (medication, dosage), lifestyle (physical activity, current or prior smoking), education, comorbidities, or the presence of radiographic erosions, correlated with phagocyte functions in RA patients. Within this context, no association was observed between phagocytosis and ROS production and any of the stated variables. Although positivity to anti-CCP and RF predicts more erosive disease [48, 49], several cohorts have reported that patients with early RA have a low frequency of joint damage during the first year of the disease [49, 50]. Because our cohort was composed of RA patients who were followed for only a short duration of the disease, it may have been too early in the course of the disease to assess significant differences in correlation between radiographic erosions and phagocytic functions. In another cohort of 101 patients, Ling at al. [51] reported that after 10.7 ± 7.9 years of disease, positivity for anti-CCP and RF had no effect on DAS-28 scores. This finding is in accordance with our results. A longer follow-up study would be necessary to thoroughly evaluate the differences in the other clinical parameters by positivity to anti-CCP and RF.

Specifically with regard to therapeutic regimens, a possible limitation for this study is that even though all patients were recruited from the same service, the drugs used in each case were not strictly the same. Nevertheless, all regimens followed the same standardized protocol [21, 22]. No association between phagocytic functions and type of therapy used (synthetic vs. biologic DMARDs) was found in this study. It is important to consider that the sample size for this study was limited and that the number of patients under biologic therapy was therefore restricted. Assessment of larger groups would yield important data to be addressed in future studies. As another limitation, our small sample size prevented many of our comparisons from reaching statistical significance, and this study did not control for many clinical and demographic variables among the patient subgroups. Another possible limitation of our study is the fact that phagocyte functions were evaluated in phagocytes derived from peripheral blood and not from synovial fluid cells, in order to evaluate directly the activation of phagocytes in the rheumatoid synovia. However, the problem is that there are several experimental concerns in study synovial cells from rheumatoid arthritis individuals in order to evaluate the activation of phagocytes without bias, because of the influence of cytokines and substances produced in consequence of the inflammatory process that is present in the rheumatoid synovia in these patients on phagocyte functions.

Our study inevitably underscores the complexity of the pathophysiological interactions between phagocytes and autoantibodies. While RA pathogenesis is complex and not completely understood, in at least some patients it is thought to depend on induction of autoimmunity in predisposed individuals who begin to produce antibodies to citrullinated antigens in the synovium. CD4^+^ T lymphocytes aid the antibody response and class switching by B cells, which results in further maturation of the anti-CCP IgG response. This B cell response that is specific for citrullinated antigens can become pathogenic once citrullinated antigens are generated in the joint and anti-CCP is able to enter the joint [52]. Our data suggest that these antibodies indirectly act on neutrophils by increasing the phagocytic capacity and superoxide anion production, possibly by enhancing the production of cytokines that up-regulate functions of the phagocytes. In addition, RF and anti-CCP may activate the classical and alternative complement system cascades, increasing the inflammatory process as a positive feedback loop [42, 53]. By means of phagocytosis and cytotoxic oxygen radical production, these activated phagocytes can initiate and perpetuate synovial damage. As memory B lymphocytes are generated, these antibodies are always being produced, therefore sustaining the lesion. This finding may explain why individuals with positive autoantibodies have more severe disease and confirms the importance of targeting phagocytes in RA therapy. The adaptive and innate immune pathways then integrate in positive feedback loops that ultimately result in a clinical picture of joint destruction and extra-articular manifestations [1–3].

To our knowledge, the present study is the first to associate the increased neutrophil phagocytosis in RA with positivity for anti-CCP. Our findings support the idea that phagocytes are precociously activated in RA, broaden understanding of the possible mechanisms by which anti-CCP and RF interact with neutrophils to cause more severe disease, and finally, suggest the importance of neutrophils and monocytes as the central effectors of tissue damage in RA.

## Conclusion

In conclusion, our data showed that overall phagocytosis and oxygen radical production by neutrophils and monocytes are enhanced in RA patients and that anti-CCP and RF positivity further enhances phagocytosis by neutrophils. Our conclusions show that measures that prevent the production of these antibodies may be a promising strategy in the management of RA.

## Abbreviations

Anti-CCP: Anti-cyclic citrullinated peptide antibodies
CR1: Complement receptor 1
CR3: Complement receptor 3
DAS-28: Disease activity score
DMARDs: Disease modifying anti-rheumatic drugs
FcγIgGr: Fcγ Immunoglobulin G receptor
HAQ: Health assessment questionnaire
IL-10: Interleukin 10
NBT: Nitro blue tetrazolium
PBS: Phosphate-buffered saline
PI: Phagocytic index
PRR: Pattern-recognition receptors
RA: Rheumatoid arthritis
RF: Rheumatoid factor
ROS: Reactive oxygen species
TGF-β: Transforming growth factor-β
Th: T helper
